# Turpentine in Post-Partum Hemorrhage

**Published:** 1890-08

**Authors:** 


TURPENTINE IN POST-PARTUM HEMORRHAGE.—E. Merwin, M. D., in *London Lancet.*—I have used spirits of turpentine in post-partum hemorrhage, and, in every case, with the best results. When the ordinary means, *i. e.*, friction over the uterus, irritation of the uterus by introduction of the fingers, cold, hypodermic injection of ergotin, etc., failed, by saturating a piece of lint with the turpentine, and introducing it with my hand into the uterus and holding it against the walls, rapid contraction took place, and all hemorrhage instantly ceased. In one or two cases, when the patient was almost pulseless, it seemed to act as a stimulant. On no occasion did its action fail, nor did it cause the slightest inconvenience, except in one, when the side of the patient's thigh was slightly blistered by some that came in contact with it, but it gave very little annoyance. I consider it to be much quicker and safer in its action than any other remedy; it does not cause any injurious result, and besides, it is much more easily applied. In country practice, getting hot water, or using injections often entails loss of valuable time.—*The Archives of Gynæcology, Obstetrics and Pædiatrics, June, 1890.*

